# Stacked Dual-Band Quantum Well Infrared Photodetector Based on Double-Layer Gold Disk Enhanced Local Light Field

**DOI:** 10.3390/nano11102695

**Published:** 2021-10-13

**Authors:** Chang Liu, Xuan Zuo, Shaohui Xu, Lianwei Wang, Dayuan Xiong

**Affiliations:** 1 Key Laboratory of Polarized Materials and Devices, School of Physics and Electronic Science, East China Normal University, Shanghai 200241, China; 51191213023@stu.ecnu.edu.cn (C.L.); shxu@ee.ecnu.edu.cn (S.X.); lwwang@ee.ecnu.edu.cn (L.W.); 2 Shanghai Key Laboratory of Multidimensional Information Processing, East China Normal University, Shanghai 200241, China; xzuo523@163.com

**Keywords:** gold disk, quantum well infrared photodetectors, localized surface plasmons, local light field enhancement

## Abstract

We propose a stacked dual-band quantum well infrared photodetector (QWIP) integrated with a double-layer gold disk. Two 10-period quantum wells (QW) operating at different wavelengths are stacked together, and gold nano-disks are integrated on their respective surfaces. Numerical calculations by finite difference time domain (FDTD) showed that the best enhancement can be achieved at 13.2 and 11.0 µm. By integrating two metal disks, two plasmon microcavity structures can be formed with the substrate to excite localized surface plasmons (LSP) so that the vertically incident infrared light can be converted into electric field components perpendicular to the growth direction of the quantum well (*E*_Z_). The *E*_Z_ electric field component can be enhanced up to 20 times compared to the incident light, and it is four times that of the traditional two-dimensional hole array (2DHA) grating. We calculated the enhancement factor and coupling efficiency of the device in the active region of the quantum well. The enhancement factor of the active region of the quantum well on the top layer remains above 25 at the wavelength of 13.2 μm, and the enhancement factor can reach a maximum of 45. Under this condition, the coupling efficiency of the device reaches 2800%. At the wavelength of 11.0 μm, the enhancement factor of the active region of the quantum well at the bottom is maintained above 6, and the maximum can reach about 16, and the coupling efficiency of the device reaches 800%. We also optimized the structural parameters and explored the influence of structural changes on the coupling efficiency. When the radius (r_1_, r_2_) of the two metal disks increases, the maximum coupling efficiency will be red-shifted as the wavelength increases. The double-layer gold disk structure we designed greatly enhances the infrared coupling of the two quantum well layers working at different wavelengths in the dual-band quantum well infrared photodetector. The structure we designed can be used in stacked dual-band quantum well infrared photodetectors, and the active regions of quantum wells working at two wavelengths can enhance the photoelectric coupling, and the enhancement effect is significant. Compared with the traditional optical coupling structure, the structure we proposed is simpler in process and has a more significant enhancement effect, which can meet the requirements of working in complex environments such as firefighting, night vision, and medical treatment.

## 1. Introduction

The development of modern technology is inseparable from the development of optoelectronic devices, and infrared photodetectors are the core of the development of optoelectronic devices [1]. At present, infrared detection plays an irreplaceable role in both military and civil affairs [2], and with the further development of modern science and technology, the application fields of infrared detectors will be more and more. The mercury cadmium telluride (HgCdTe) infrared detector [3] is the first-generation infrared detector with high quantum efficiency and can be used normally at room temperature. It is the most common infrared detector in daily life. With the development of semiconductor technology, quantum well infrared photodetectors (QWIP) has gradually matured in various fields. QWIP has many advantages, such as high stability, low cost, and simple process. However, according to the quantum transition selection rule, the vertical incoming infrared light cannot be absorbed by the quantum well, so the coupling structure is needed to change the direction of the infrared light [4,5]. The main coupling modes are edge coupling, grating coupling, random reflection coupling, and microcavity coupling [6,7]. With the continuous improvement of QWIP performance, the requirements for coupling devices are also increasing, so how to increase the photoelectric coupling efficiency is still one of the main challenges of current research [8,9,10,11,12,13].

Metamaterials are special materials manufactured artificially and do not exist in nature. The electromagnetic properties of traditional materials are determined by their physical and chemical composition, but metamaterials can change the electromagnetic properties by changing the shape, size, and period of the structure. The general metal-insulator-metal (MIM) metamaterial structure can achieve higher absorption, but the absorption peak is single and narrow and cannot achieve multi-band and broadband detection [14,15]. In 2019, Liu et al. proposed to use the titanium nanoholes meta-surface to make an ultra-wideband infrared absorber. There are two absorption peaks at 1.30 μm and 2.56 μm, and the absorption rate is above 90%. The average absorption rate in the 1–3 μm band is as high as 80% [16]. Zhou et al. have made an ultra-wideband infrared absorber that works at 8–14 μm by integrating titanium nano-ring structures and titanium nano-antennas in a working unit [17]. In addition to integrating metals with different structures on the surface, the stacked metamaterial can also be used to enhance the absorption of dual-band QWIP. For example, Hou et al. have designed a stacked metamaterial absorber to achieve absorption of mid-wave infrared and long-wave infrared. There are two strong absorption peaks at 3.89 and 9.97 μm, and the absorption rate is as high as 99% [18]. Unlike traditional photoelectric coupling devices, novel metamaterials structures can not only increase local optical field coupling but also achieve multi-band optical absorption. At present, metamaterials are often used in infrared photoelectric devices for perfect absorption in infrared bands [19,20]. Integrating metamaterials into infrared photodetectors to enhance photoelectric coupling will become an important way to improve the performance of detectors [21].

The classic dual-band quantum well infrared photodetector is generally a double-layer stacked structure. In 2020, Hou et al. reported a metamaterial infrared absorber with a stacked structure [18]. So, we optimized the infrared absorber and integrated it into the dual-band quantum well infrared photodetector. In this work, we have proposed a stacked dual-band QWIP integrated with a double-layer gold disk. Two 10-period quantum well layers are stacked together, and gold nano-disk structures are integrated on their respective surfaces to enhance the local electric field to form two MIM structures. The device we designed works in the two infrared bands of 13.2 and 11.0 μm, and the electric field is obviously enhanced at these two wavelengths. Through our designed structure, the incident infrared light can be coupled into the active region of the quantum well. We optimized the structural parameters to obtain the best electric field enhancement at different wavelengths so that we can maximize the performance of the device at the wavelength of interest.

## 2. Materials and Methods

**Figure 1:**
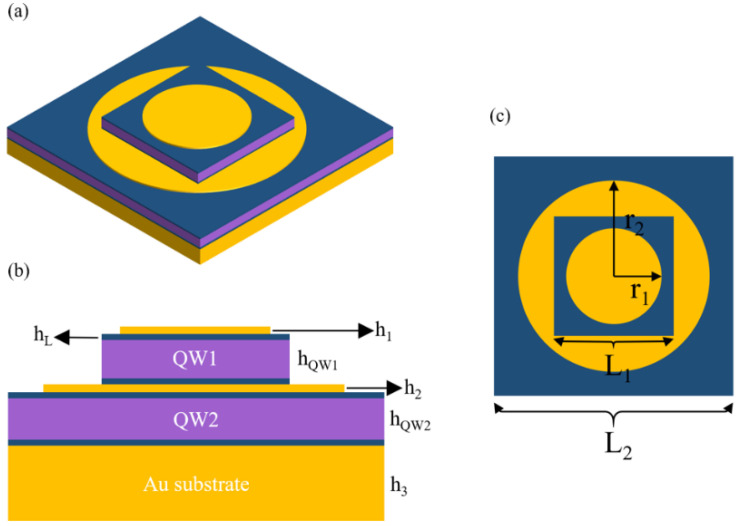
Schematic diagram of double-band stacked quantum well infrared photodetector structure: (**a**) the three-dimensional view, (**b**) the unit cell from the x-z plane view; (**c**) the top view.

The device structure we proposed is shown in Figure 1. Figure 1a shows the three-dimensional structure of the device. Figure 1b,c are the front view and top view of the device, respectively. The QWIP layer grown by molecular beam epitaxy on a GaAs substrate could be transferred to an Au substrate by wafer bonding [9]. The device is composed of two MIM structures, and the widths of the two quantum well active regions are, respectively, L_1_ and L_2_ (L_1_ = 2.5 μm, L_2_ = 5 μm). The first quantum well active region (QW1) consists of 10 periods GaAs/Al_0.20_Ga_0.80_As. The well width is 5.3 nm, and the barrier width is 50 nm. The thickness of the first quantum well active region is defined as h_QW1_, sandwiched between two contact layers with a thickness of h_L_ (h_L_ = 75 nm). The contact layer is GaAs layers with 10^18^ cm^−3^ Si donors. On the top surface of the device, there is a nano-disk structure made of Au with a thickness of h_1_(h_1_ = 0.1 μm) and a radius of r_1_(r_1_ = 1 μm). The second quantum well active region (QW2) consists of 10 periods GaAs/Al_0.25_Ga_0.75_As. The well width is 4.6 nm, and the barrier width is 50 nm. Similarly, the thickness of the second quantum well active region is defined as h_QW2_, sandwiched in the contact layer. Between the two quantum well regions, there is also a gold nano-disk with a thickness of h_2_(h_2_ = 0.1 μm) and a radius of r_2_(r_2_ = 2 μm). We choose to use gold with a thickness of 1 μm as the substrate to prevent transmission and build a MIM structure. The relationship between absorption wavelength and energy is as follows [22]:

(1) $$\lambda =\frac{1.24}{E\left(eV\right)}$$

**Figure 2:**
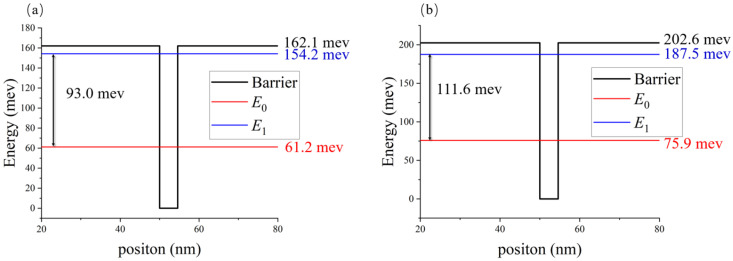
Energy level diagram inside a quantum well: (**a**) quantum well energy level operating at a wavelength of 13.2 μm (QW1) and (**b**) quantum well energy level operating at a wavelength of 11.0 μm (QW2).

The device we designed has absorption peaks at 13.2 μm and 11.0 μm. Therefore, by adjusting the aluminum composition of AlGaAs and the well width and barrier width of the quantum well, the energy level of the quantum well we selected is shown in Figure 2. Figure 2a shows the energy level diagram of a single quantum well with a detection wavelength of 13.2 μm, in which the bound state energy level is 162.1 mev, the ground state energy level is 61.2 mev, and the first excited state energy level is 154.2 mev. Similarly, Figure 2b shows the energy level diagram of a single quantum well with a detection wavelength of 11.0 μm, in which the bound state energy level is 202.6 mev, the ground state energy level is 75.9 mev, and the first excited state energy level is 187.5 mev.

In the preparation of GaAs/AlGaAs multi-quantum well materials, the control of the material cycle thickness and material components is very important in order to prepare the material with target components and target cycle thickness, which provides the basis for the later device development. Therefore, we need to characterize and test the grown materials. Rapid thermal annealing (RTA) not only effectively removes defects and dislocations from the material but also induces more uniform interfacial mixing, thus improving the quality of the material and the performance of the device. We made an error analysis of the Al composition and well width of the quantum well. We floated the Al composition up and down by 0.02, and the well width error was 0.1 nm to obtain the following changes in energy level and detection wavelength:

For QW1, when the Al component is shifted up or down by 0.2, the detection wavelength varies between 11.02 and 17.08 μm. When the trap width is shifted by 0.1 nm, the detection wavelength varies between 12.58 and 13.75 μm. Similarly, for QW2, when the Al component is shifted by 0.2, the detection wavelength varies from 9.68 to 14.6 μm, and when the trap width is shifted by 0.1 nm, the detection wavelength floats between 10.72 and 12.74 μm.

## 3. Results and Discussion

**Figure 3:**
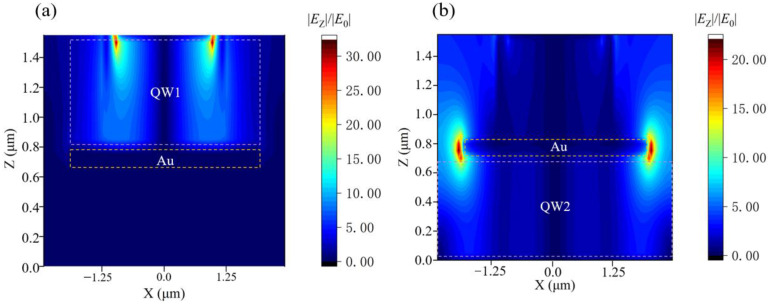
**|***E*_Z_**|**/**|***E*_0_**|** distributions of the two quantum well layers in the *x-z* plane (*y* = 0) at 13.2 and 11.0 μm: (**a**) **|***E*_Z_**|**/**|***E*_0_**|** distribution of the quantum well working at 13.2 μm, (**b**) **|***E*_Z_**|**/**|***E*_0_**|** distribution of the quantum well working at 11.0 μm, the purple dashed line indicates the active region of the quantum well.

Figure 3 shows the electric field distribution in the *x*-*z* plane (*y* = 0). We used the FDTD method to carry out the numerical simulation calculation of the *E*_Z_ electric field [23]. In this paper, periodic boundary conditions are used for both x and y directions of the model, and perfect matched boundary conditions are used for the z direction to ensure complete absorption of light energy. The incident wave is adopted as a plane wave along the negative direction of the z-axis, and the electric field component is along the z-axis direction with an intensity of 1 V/m. The two GaAs layers and the middle QWs layer correspond to a homogeneous dielectric layer with a refractive index of 3.3 [24]. We divide the mesh into 0.05 μm into the x and y directions and 0.06 μm in the z direction. Figure 3a shows that at the incident wavelength of 13.2 μm, the electric field in the active region of the first quantum well at the top is enhanced by the metal on the surface, which is increased by up to 20 times compared with the **|***E*_0_**|** of the incident light. Figure 3b shows the enhancement of the local electric field by the gold disk in the middle at the incident wavelength of 11.0 μm, and the enhancement of the electric field in the active region of the second quantum well at the bottom, which is similarly increased by a maximum of about 20 times compared to the **|***E*_0_**|** of the incident light. It is four times stronger than that of the gold 2DHA reported in reference [25]. In reference [13], the author proposed metallic optical incouplers to enhance the *E*_Z_ electric field in the active region of the quantum well. Using QWIP with metallic optical incouplers, the maximum *E*_Z_ electric field enhancement in the active region of the quantum well is 10, and the structure we proposed is about two times that of the metallic optical incouplers.

**Figure 4:**
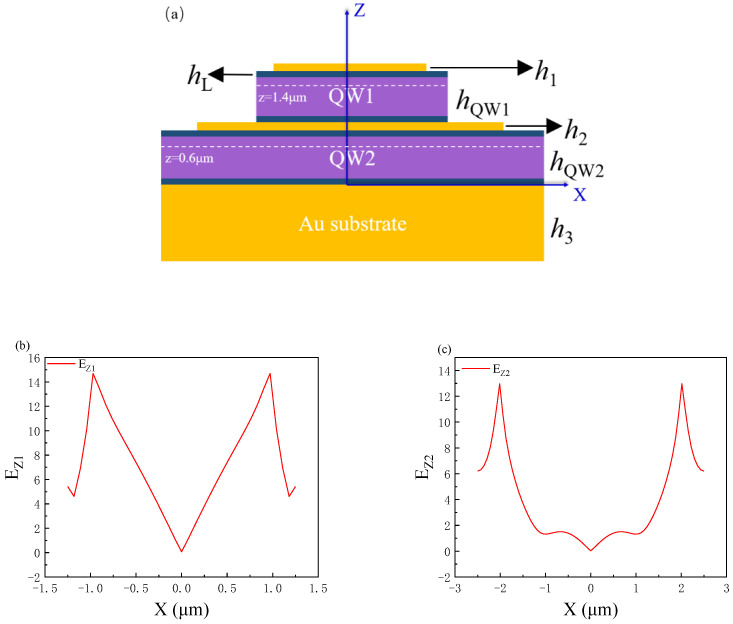
(**a**) At the *x-z* plane (*y* = 0) of the device, take the zero point at the center of the device at the metal-semiconductor interface to establish the coordinates. (**b**) The electric field component *E*_Z_ in QW1 changes with the x-axis (*y* = 0, *z* = 1.4 μm) and (**c**) the electric field component *E*_Z_ in QW2 changes with the x-axis (*y* = 0, *z* = 0.6 μm).

To demonstrate the distribution of the *E*_Z_ electric field more visually, we make the interface between the gold substrate and the QW2 as the zero point of the coordinate z-axis, as shown in Figure 4a. Figure 4b shows the electric field enhancement distribution of 0.1 μm from the metal in the first quantum well layer on the top (*y* = 0, *z* = 1.4 μm). It can be seen from the figure that there is a strong electric field enhancement on both sides of the metal disk near the metal-semiconductor interface at the incident wavelength of 13.2 μm, and the electric field does not extend laterally to the middle but has a deep expansion in the *z* direction. This is consistent with the characteristics of the localized surface plasmons [26], which proves that the MIM structure excites the localized surface plasmons and enhances the electric field in the active region of the quantum well. Figure 4c shows the electric field distribution of the second quantum well active region at the bottom at the same position (*y* = 0, *z* = 0.6 μm). Like the first quantum well layer at the top, the electric field in the active region of the quantum well is enhanced at an incident wavelength of 11.0 µm when the local surface plasmon excitations are excited. For a traditional single surface coupling device, the enhancement effect of the bottom will continue to attenuate, and for the stacked quantum well structure, the enhancement effect of the active region of the bottom quantum well will be insignificant. Unlike the traditional surface coupling device, when the metal disk in the middle excites the LSP, the active region of the quantum well at the bottom can also be effectively enhanced.

Since the SPP effect shows a decaying effect, the further away from the metal-semiconductor interface, the worse its enhancement effect is, and some even have no enhancement effect, so we need to calculate the *E*_Z_ of the whole quantum well active region to analyze whether the whole quantum well active region has an enhancement effect. For QWIPs devices, each plane in the active region of the quantum has the enhancement of the *E*_Z_ electric field component is a perfect effect. However, the **|***E*_Z_**|**^2^ generated at the metal-semiconductor interface shows an exponential decay along the negative direction of the z-axis [27]. To probe **|***E*_Z_**|**^2^ across the active region of the quantum well, according to the definition F is as follows [27]:

(2) $$F=\frac{\iint_{Z=S}{\left|E_{Z}\left(x,y\right)\right|}^{2}dxdy}{\iint_{Z=S}{\left|E_{0}\left(x,y\right)\right|}^{2}dxdy}\;$$

where *S* is the coordinate of the z-axis and *E*_0_ is the intensity of the incident light.

**Figure 5:**
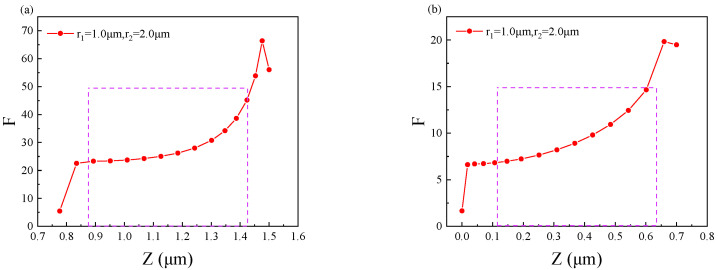
The variation of the enhancement factor F in the two quantum well layers with the coordinates Z: (**a**) variation in QW1 and (**b**) variation in QW2.

Figure 5 shows the enhancement factor *F* for the active regions of the two quantum wells as a function of the z-axis. The purple dotted line in the figure indicates the active region of the quantum well. Figure 5a shows the enhancement factor *F*_1_ for the QW1, which working at a wavelength of 13.2 μm. Figure 5b shows the enhancement factor F_2_ for the QW2, which working at the wavelength of 11.0 μm. It can be seen from the figure that *F* decreases as the distance to the metal-semiconductor interface increases. However, in the quantum well active region, *F* still has high values, the maximum value of *F*_1_ in QW1 is 45, and the maximum value of *F*_2_ in QW2 is 16. *F*_1_ always remains above 25 in the quantum well active region, while *F*_2_ also remains above 6. The above proves that our structure can guarantee that the electric field is always enhanced in the designed length of the active region.

**Figure 6:**
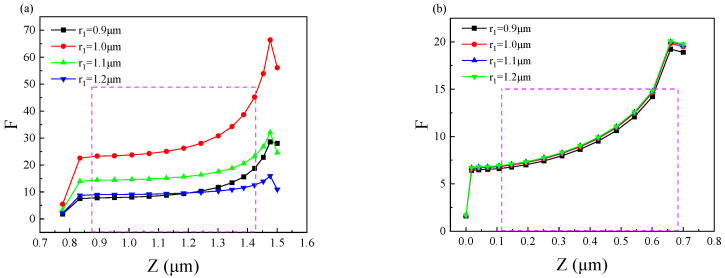
The effect of changing the r_1_ of the top metal disk on the enhancement factor: (**a**) the effect of different r_1_ on the enhancement factor of the top quantum well layer (QW1) and (**b**) the effect of different r_1_ on the enhancement factor of the bottom quantum well layer (QW2).

Next, we optimize the size of the gold disk. Figure 6 shows the effect of a top metal disk with a different radius(r_1_) on the enhancement factor of the device. As can be seen in Figure 6a that different r_1_ mainly affects the top quantum well layer (QW1). At the incident infrared wave wavelength of 13.2 μm, the enhancement factor reaches the maximum when r_1_ = 1.0 μm. It can be seen from the figure that if r_1_ is not 1.0 μm, the enhancement factor will decrease whether r_1_ increases or decreases, so r_1_ = 1.0 μm can achieve the best performance. It can be seen from Figure 6b that for the bottom quantum well layer (QW2), the change of r_1_ has little effect on the enhancement factor, which remains almost constant.

**Figure 7:**
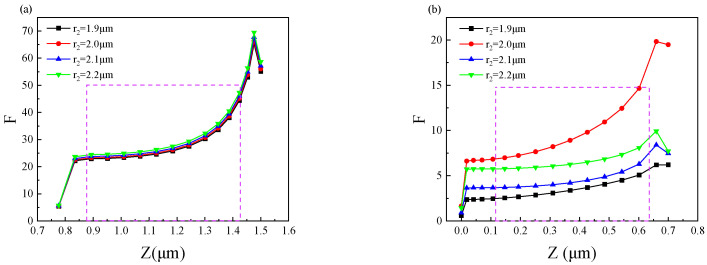
The effect of changing the r_2_ of the middle metal disk on the enhancement factor: (**a**) the effect of different r_2_ on the enhancement factor of the top quantum well layer (QW1) and (**b**) the effect of different r_2_ on the enhancement factor of the bottom quantum well layer (QW2).

Figure 7 shows the effect of changing the radius(r_2_) of the intermediate metal disk on the enhancement factor of the device. Figure 7a shows that the effect of varying r_2_ on the enhancement factor is not significant for the top quantum well layer (QW1) at an incident wavelength of 13.2 μm. It can be seen from Figure 7b that taking r_2_ = 2.0 μm at the incident infrared wavelength of 11.0 μm has the largest enhancement factor for the bottom quantum well layer (QW2). In summary, for the double-layer metal disk structure we designed, the best enhancement effect can be obtained at r_1_ = 1.0 μm and r_2_ = 2.0 μm.

For QWIPs, the photocurrent is proportional to the average |*E*z|^2^ of the whole active region. To describe the infrared absorption of the dual-band QWIP with the double-layer gold disk, we calculated the coupling efficiency *η* according to the following definition:

(3) $$\;\eta =\frac{{∭}_{active\;layer}|E_{Z}\left(x,y,z\right){|}^{2}dxdydz}{{∭}_{active\;layer}|E_{0}\left(x,y,z\right){|}^{2}dxdydz}$$

where **|***E_0_***|**^2^ is the intensity of incident light, which is 1 (V/m)^2^ in simulation.

**Figure 8:**
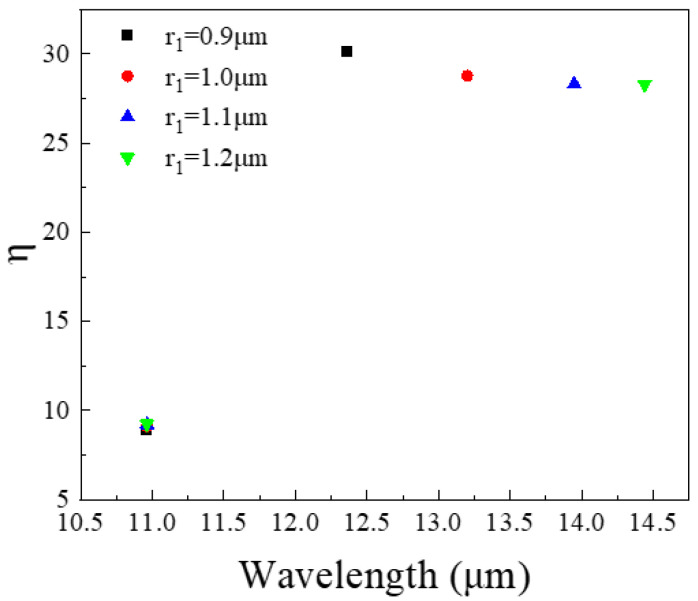
In the wavelength range of 10.5–15 μm, the maximum coupling efficiency corresponding to different r_1_.

Figure 8 shows the effect of different r_1_ on the maximum coupling efficiency in the wavelength range of 10.5–15 μm. The data points in the figure represent the maximum coupling efficiency at different wavelengths. It can be seen from the figure that when r_1_ increases, the maximum coupling efficiency of the top quantum well layer (QW1) working at an incident infrared wavelength of 13.2 μm will shift toward a longer wavelength. Therefore, as the increase in r_1_, the wavelength corresponding to the maximum value of the coupling efficiency is also increasing. For the bottom quantum well layer (QW2), the maximum coupling efficiency does not change with the change of r_1_ because r_1_ only affects the coupling efficiency of QW1.

**Figure 9:**
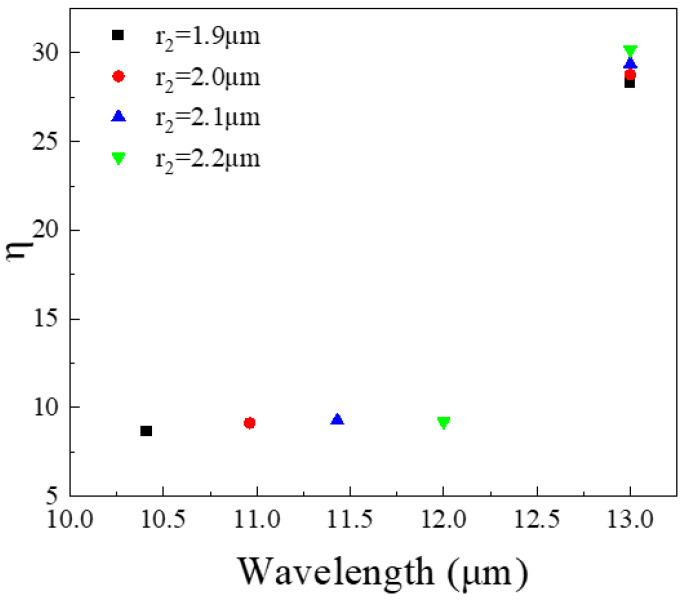
In the wavelength range of 10.5–15 μm, the maximum coupling efficiency corresponding to different r_2_.

Figure 9 shows the effect of different r_2_ on the maximum coupling efficiency of the bottom quantum well layer (QW2). We set r_2_ to increase from 1.9 to 2.2 μm, the maximum coupling efficiency of QW2 will also shift at long wavelengths as the wavelength increases. It has little effect on the top quantum well layer (QW1). In summary, we can optimize the radius of the disk to locate the maximum coupling efficiency at different wavelengths.

## 4. Conclusions

In conclusion, we have numerically simulated the proposed stacked dual-band quantum well infrared photodetector based on a double-layer gold disk-enhanced local light field using the FDTD method. This structure can make QWIP have strong electric field enhancement at the incident wavelengths of 13.2 and 11.0 μm. The LSP is excited by two different gold disks to enhance the electric field in the quantum well layer. The maximum is about 20 times compared to the incident light. We have also simulated the influence of the disk radius on the enhancement factor and coupling efficiency. By optimizing the radius of the disk, we can determine that r_1_ = 1.0 μm, r_2_ = 2.0 μm can make the device obtain the best enhancement factor. As the radius of the disk is increased, the wavelength corresponding to the maximum value of the coupling efficiency also increases, resulting in a red shift. The double-layer metal disk structure designed by us can effectively enhance the local electric field *E*_Z_ of the stacked dual-band QWIP, improve the enhancement factor F and coupling efficiency η of the quantum well region, which is of great help to the performance enhancement of the dual-band QWIP.

## Data Availability

The data presented in this study are available on request from the corresponding author.
